# Impact of digital wound care solution on healing time: A descriptive study in home health settings

**DOI:** 10.1371/journal.pdig.0000855

**Published:** 2025-05-30

**Authors:** Heba Tallah Mohammed, Robert D. J. Fraser, Amy Cassata

**Affiliations:** 1 Swift Medical Inc., Toronto, Ontario, Canada; 2 Arthur Labatt School of Nursing, Western University, London, Ontario, Canada; Instituto Politécnico Nacional Escuela Superior de Medicina: Instituto Politecnico Nacional Escuela Superior de Medicina, MEXICO

## Abstract

**Background:**

Chronic wounds pose significant challenges in home healthcare (HH) due to prolonged healing times and high costs. Digital wound care solutions (DWCS) have shown potential for improving healing efficiency. This study evaluated the impact of continuous DWCS use on healing times at HH organizations and explored area reduction in non-healed yet improved pressure injuries (PIs) and diabetic ulcers (DUs).

**Methods:**

This descriptive study analyzed 195,915 wound assessments from 59 HH organizations using DWCS in 2022 and 2023. Average healing time was calculated by wound type and compared across the two years, with subgroup analyses for wounds healing within three months versus longer. Improvements in non-healed DUs and PIs were further categorized by initial wound size (≤2 cm², >2 cm² for DUs; ≤4 cm², >4 cm² for PIs).

**Results:**

Average healing time for all wounds decreased significantly from 62.5 days in 2022 to 38.6 days in 2023, a 38.2% improvement (p < 0.001). DU and PIs showed reductions of 30.8 and 29.3 days, respectively. The proportion of wounds healing within three months rose by 8.9%, with decreased average healing times within this period. For wounds requiring over three months, the average time saved was 57.6 days (8.2 weeks; P = 0.014), representing a 27% improvement. Non-healed but improving PIs showed increase in area reduction from 5.2 cm² to 17.7 cm², with a 25.4% faster time to reduction. Larger PIs (>4 cm²) showed greater reductions, with time to improvement decreasing by 35.5 days (34.7%, p < 0.001). DUs also improved, with area reduction increasing from 4.8 cm² to 15.3 cm² and a 23.8% faster reduction time, while larger DUs (>2 cm²) saw a 32.6-day decrease in time to improvement.

**Conclusion:**

Continuous DWCS use significantly reduces healing times and improves wound area reduction, underscoring its effectiveness in enhancing wound care outcomes in HH settings.

## Author summary

This descriptive research evaluated the outcome of the ongoing use of a Digital Wound Care Solution (DWCS) on wound healing and area reduction within home health agencies (HHAs). Data evaluated from 195,915 wound assessments across 59 HHAs between 2022 and 2023 showed a significant improvement in average healing times by 38.2% (from 62.5 days to 38.6 days). Specifically, diabetic ulcers (DUs) and pressure injuries (PIs) demonstrated notable reduction (30.8 days for DUs and 29.3 days for PIs). Furthermore, the overall proportion of wounds successfully healed within three months improved by 8.9%, and the average time saved for wounds taking greater than three months to heal had an overall savings of 57.6 days. Non-healing wounds with improvement also achieved significant area reduction, with the area of PIs improved from 5.2 cm² to 17.7 cm² (25.4% faster), and DUs improved from 4.8 cm² to 15.3 cm² (23.8% faster). Larger wounds showed a decrease of over 30 days in time to improvement. These study findings highlight the impact of DWCS in improving the healing and management of chronic wounds, especially DUs and PIs, in home health settings.

## Introduction

Due to factors such as an aging population and increasing comorbidities like obesity and diabetes, the United States is currently witnessing a significant rise in the incidence of chronic wounds [1,2]. According to data derived from the Medicare database, it is estimated that at least 6.5 million patients in the US are living with chronic wounds, making it a major public health concern [1,2].

Diabetic ulcers (DU) ranked as the most common cause of chronic wounds, affecting approximately 10% of diabetes patients in the USA [3]. Studies have shown that the prevalence of foot ulcers among diabetics is higher compared to other diabetic complications like ischemic heart disease, cerebrovascular disease, and retinopathy [4,5]. With the annual incidence of type 1 diabetes at 2.6% and 3% for type 2 diabetes [6,7], there is a 19–34% higher risk of developing diabetic foot ulcers throughout the lifetime [8].

Following diabetes, venous ulcers (VU) are the second most common cause of chronic wounds, accounting for approximately 15–20% of cases observed in vascular and foot care clinics [9]. In addition, pressure injuries (PI) affect an estimated 2.5 million individuals each year in the United States alone [10]. The prolonged healing and associated complications of these prevalent chronic wounds pose a significant financial burden to the healthcare system, with expenditures exceeding $25 billion USD per year [2]. This is due to the substantial consumption of healthcare resources and ongoing nursing care for wound management [11,12].

On average, chronic wounds require up to 50% more dressings than acute ones to promote healing, and increased dressing change frequency is associated with higher turnover rates, placing additional strain on healthcare resources [13]. The existing reimbursement policies related to wound care often lack the necessary specificity, inadvertently encouraging costly treatment methods that may not optimize patient outcomes [14]. Therefore, in the case of chronic wounds, it is important to track the healing progress and understand the underlying causes of failure to progress in a timely fashion through the wound healing stages and to turn a chronic wound into a healing wound [15].

A comprehensive approach to both assessment, management and documentation, utilizing interdisciplinary expertise, is essential [16]. Wound assessment should encompass a detailed evaluation and documentation of the wound bed status, characteristics, precise wound measurements, and pertinent local and systemic factors, including any existing comorbidities [17]. Comprehensive understanding of a patient’s wounds and overall health condition enables the determination of wound healing trajectories, which are vital for developing high-quality treatment strategies and ultimately achieving wound healing [16]. However, Ding and colleagues’ literature review study highlighted the limited evidence on wound assessment and documentation, particularly in relation to the frequency of wound assessments and the extent to which these assessments are documented [18].

Therefore, the healthcare sector faces significant challenges. The limited understanding and variability in documented wounds and their healing progress across different types of chronic wounds makes it challenging to accurately predict the necessary resources for effective treatment, leading to inefficiencies in care delivery [19,20]. Moreover, estimating the precise costs associated with specialized care becomes increasingly difficult, as the unpredictability of healing times can result in unexpected financial burdens for both healthcare providers and patients [14]. Additionally, the lack of clarity regarding healing durations hinders the development of comprehensive management plans tailored to individual patient needs and can lead to increased anxiety and dissatisfaction among patients [21]. Therefore, acquiring more robust and comprehensive data on the healing progress and times of different chronic wounds is critical for improving patient outcomes, enhancing resource allocation, and optimizing care strategies in healthcare settings [11,20].

Ousey and colleagues descriptive study highlighted significant variability in beliefs and an inconsistent application of therapeutic decisions during wound care assessments [17]. This was evident when they surveyed attendees of the European Wound Management Association (EWMA). They found that only 60% of the respondents reported utilizing established frameworks for conducting wound bed assessments in their clinical practice. This finding is particularly concerning given the respondents’ awareness and understanding of the importance of wound assessment tools, which suggests a gap between knowledge, wound assessment and practical application in wound care management [17].

Therefore, many healthcare organizations started leveraging artificial intelligence (AI) powered digital assessment tools to support a standardized assessment approach [20]. Evidence supports the efficacy of using AI technology in wound care assessment. For example, a quasi-experimental study evaluated an AI-based wound assessment tool’s usability and efficacy and provided evidence of the potential of AI in the field of wound management. Results indicated the application enhanced wound evaluation, facilitated care plan sharing, boosted patient compliance, and improved virtual care efficiency. The study demonstrated the potential of AI in wound management [22].

Therefore, digital tools designed to consistently guide clinicians through the assessment process would ultimately lead to effective treatment decisions [17]. Consistency and standardization of assessments using the digital tools are the key to optimizing patient outcomes and ensuring that care is both effective and evidence based [17].

**Fig 1:**
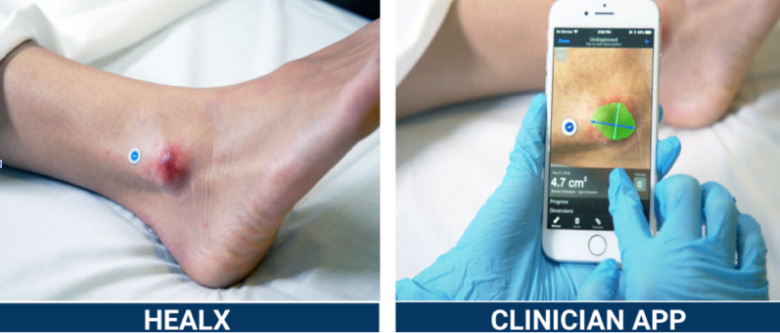
FDA-approved reference marker and capturing wound image.

Swift Skin & Wound (Swift Medical Inc., Toronto, Canada) is a non-intrusive digital wound care solution (DWCS) employing AI for standardized wound evaluation. The DWCS utilizes an FDA-approved reference marker (Fig 1) to obtain scientifically calibrated, color-corrected images and employs intelligent algorithms to automatically delineate wound margins, measure dimensions, precisely calculate surface area, depth and instantly upload and document this data in patients’ medical records [23]. Research shows a 99% measurement consistency among clinicians using Swift, ensuring reliability across experience levels [24]. The clinician-facing dashboard allows interdisciplinary teams to monitor patient progress, coordinate care, and offer real-time recommendations. Further, it provides detailed documentation that can protect clinical decisions, assist compliance with laws, and lower healthcare liability [20]. DWCS uses computer vision and deep learning algorithm to detect and accurately quantify the types of tissue in a wound, regardless of skin tones and the Healing Index draws on AI-based modeling and prediction to analyze wound characteristics and project healing trajectories for better treatment planning. Studies demonstrate that Swift Skin & Wound’s wound analytics, automation, and documentation approach significantly improve operational and clinical outcomes. It reports a significant reduction in days to heal for pressure injuries [20] and saving clinicians a significant amount of time in assessing wounds [23]. Additionally, it accurately measures wound dimensions irrespective of variability in wound bed characteristics or skin tone [25].

This study represents the first systematic effort to document wound healing times using the DWCS (Swift Skin and Wound). We utilized the DWCS’s large, clinically validated database in a home healthcare (HH) environment. The primary goal was to accurately determine the average duration for wound healing in the HH setting and analyze changes in healing times over time, specifically in 2022 and 2023, with the consistent use of this innovative technology.

In addition to the primary focus, the study also compared the average healing times and the proportion of wounds that achieved healing after using the solution, categorized by their respective healing durations. This included a specific examination of wounds that healed in less than three months compared to those that took longer than three months in the assessment years (2022 and 2023). The research also explored the proportion of area reduction, and the percentage of improvement observed in non-healed but improved diabetic ulcers (DU) and pressure injuries (PI) within the same assessment years (2022 and 2023).

## Methods

### Study design and data sources

The descriptive study analyzed the change in average healing times for various wound types across a cross-sectional sample of 59 home health agencies (HHA) that integrated the solution in 2021 and maintained DWCS as part of their wound care program in 2022 and 2023.

Some agencies were located in various geographical regions of the United States, which enhances the generalizability of the findings in this study. Included in the sample are agencies from Northeastern, Midwestern, Southern, and Western parts of the United States. Through sampling from all four regions, the attempt was made to capture variations that exist in local infrastructure for providing health care services, payment programs, and population demographics. Of the agencies, about 40% are situated in urban areas, 35% in suburban areas, and 25% in rural areas, thus ensuring balanced representation of home health settings.

Agencies varied greatly in their size and operational scale, given that they spanned from small agencies with average daily census of 50–100 patients (40%), medium size agencies (35%) with 100–500 patients, and large ones (25%) with over 500 patients across various states. This variation contributed, among other things, to differences in allocation of resources, workforce capacity, and technological integration. Most small agencies operate with small teams of 10–20 clinicians. In contrast, larger agencies could have more than 100 field staff which could include nurses, physical therapists, occupational therapists, home health aides, and case managers.

Different staffing models and types of workflows represented in home care were also reflected in this study. Thus, some agencies employ the traditional clinician-based model where registered nurses and physical and occupational therapists provide direct supervision of patient care. In contrast, other agencies use a case management approach where a primary clinician coordinates care among interdisciplinary teams.

The solution software, user training, and technical support are standardized across all HHAs, facilitating a standardized utilization process across different HHAs. Additionally, the study examined the average healing times and proportion of healed wounds categorized by their healing duration (wounds that healed in less or more than three months). It also explored the proportion of area reduction and percentage of improvement for non-healed but improved diabetic ulcers and pressure injuries.

The study utilized the large, clinically calibrated and validated DWCS database specifically designed for gathering and securely storing anonymous wound care data from participating HHAs. Data collected from the application is gathered from clinicians’ standard patient care wound assessments at the participating HH organizations that adopted the solution. The system’s unique feature is its generation of a unique code for each patient whenever a wound is assessed, which serves as the identifier for storing the wound data. This advanced system then can either retrieve the direct patient identifiers from the app of the digital solution for the site’s follow-up purposes or purge them, thus providing an anonymous dataset for research purposes. This ensures the meticulous and confidential collation of comprehensive wound information.

The server communications are encrypted using the Advanced Encryption Standard. Our secure database strictly adheres to privacy standards outlined in various acts, including the Health Insurance Portability and Accountability Act (HIPAA). The data warehouse engineers maintain the anonymous analytic database, and the clinical innovation team has secure administrative access. This systematic, secure collection of anonymized data facilitates comprehensive analysis for research purposes. This process does not alter or influence the care administered to the patient.

The DWCS database compiles a wide array of clinical and administrative data, encompassing patient assessment details such as unique ID code, age, sex, wound evaluation dates, wound type and class, anatomic location of the wound, temperature, odor, Bates-Jensen Wound Assessment Tool (BWAT) score, pain level, wound status progress, wound acquisition status (whether external or after admission), wound measurements including length, width, surface area, and depth. Additionally, the database includes evaluation review history (sign and lock progress), evaluation linked with images, drainage usage, and details of the role of clinicians conducting evaluations along with the healthcare settings and corresponding organizational branches.

This descriptive quality improvement study obtained an exemption for ethics review from Pearl IRB, LLC, an independent institutional review board (ID: 2023–0100). The exemption was granted due to the study using only fully de-identified, retrospective wound assessment data collected in the normal clinical setting, with no direct patient contact or impacts on treatment or decisions. Each organization participating in the study was made known that anonymized data may be used for quality improvement and research purposes. The digital health platform uses end-to-end encryption, stringent access controls, and compliance with HIPAA and other applicable privacy laws to ensure data privacy and security. Other robust de-identification protocols are in place to remove personally identifiable information that could lead to re-identification before data extraction and analysis. Such procedures uphold the ethical principles in the Declaration of Helsinki, ensuring appropriate handling of sensitive health data with an opportunity for advancements in evidence-based wound care.

### Data abstraction process and sample

The anonymous wound care assessment records were collected from the DWCS database. The clinical innovation research team accessed the DWCS database and extracted all required wound patient data based on the wound start data from January to March 2024.

To ensure methodological rigor and data integrity, a structured data abstraction and validation process was employed. Data were extracted from the DWCS database. The research team performed structured queries to retrieve all relevant wound assessment records from participating 59 home health agencies (HHAs). Multiple levels of quality control were enacted to ensure accuracy, consistency, and completeness of the dataset.

#### 1. Data validation process.

The DWCS system has built-in validation checks that minimize errors in data entry, thus ensuring that the recorded wound assessments meet standardized documentation criteria. Additionally, the system also imposes a structured workflow by requiring mandatory fields to be filled out before allowing data-entry submission, which should avoid possible missing values in key fields, e.g., wound type, classification, and status progress. During data extraction, audits were performed regularly on the data to ensure that they were accurately transcribed and consistently formatted across different HHAs.

#### 2. Handling of missing data.

To manage missing data, a data completeness assessment was performed by the research team, ensuring that each record with missing or incomplete data had been examined systematically. Records containing missing key variables were excluded from the corrected analysis to avoid introducing bias into subsequent comparisons of outcomes. Since the size of the dataset (195,915 wound assessments) rendered imputation unnecessary, the presence of a sufficient volume of complete cases allowed for reliable statistical analysis.

#### 3. Inter-rater reliability measures.

The inter-rater reliability is sustained through standardized documentation protocols and systematic training of clinicians using the DWCS technology. The solution software, user training, and technical support are also standardized across the HHAs to facilitate a consisted utilization.

To be eligible for inclusion in the study a patient’s wound evaluation data must have fulfilled the following criteria:

1. Wound records (primary and secondary diagnoses) of any type were assessed at the participating 59 HHAs during the study period (2022 and 2023).
2. Wounds had to be assessed and managed using the DWCS technology
3. The records pertained to adult patients 18 or older.

Any wounds outside of the study period were excluded from the analysis. Wounds assessed at adoption branches other than the participating 59 HHAs were not included in the analysis. The study gathered and incorporated data from 195,915 wounds assessed at the participating HHAs during the study periods in 2022 and 2023. Specifically, 66,878 wounds were assessed in 2022, and 129,040 wounds were assessed in 2023.

Each patient in the study was assigned an ID number to ensure anonymity, and no medical record number (MRN) was accessed. The study aimed to compare clinical outcomes over time by analyzing the change in the average/median days to heal a wound from 2022 to 2023 for the 59 HHAs. The study extracted the following indicators from the eligible wound assessments for analysis in this study: a) wounds’ evaluation dates, b) patient demographic characteristics such as age and sex, c) wound status progress (new, worsened, improving, closed/healed), e) wound acquisition status (whether external or after admission), f) wound type and class and, g) wound surface area.

Using the wound evaluation date metric, the study generated the first and last dates of evaluations. Days to heal were calculated as the time lapse between the first and last assessment date for the wounds marked as healed by the clinician. The study also categorized wounds into two groups based on the time lapse between the first and last evaluation for wounds marked as healed: wounds healed in < 3 months and wounds healed in > 3 months. A comparison of the proportion and the average days to heal the wounds that healed in less than three months and those healed in more than three months across all wounds was conducted.

Furthermore, the study investigated non-healed but improved diabetic ulcers (DUs) and pressure injuries (PIs). The study analyzed PI and DU wounds, which showed improvement and decreased surface area size. It examined the average reduction in area size, the change in the average time to recorded improvement, and the percentage of improvement between 2022 and 2023. The study also stratified the data for the same study period and for the same wound types based on the initial size of the DUs and PIs surface area, subcategorizing each PI wound based on the initial size of <= 4 cm^2^ and > 4 cm^2^ and DUs based on the initial size of <= 2 cm^2^ and > 2 cm^2^.

This study utilized a clinically validated benchmarks to define “improved but not healed” wounds. This improvement is defined by the percentage reduction in wound surface area over time and is widely recognized within the wound-healing research community. If a wound showed a 40–50% decrease in surface area between the first and last recorded assessments of a patient during the study period, it was considered “improved.” This cut-off point was forged from previous studies indicating that a closing of the wound size by 40–50% within four weeks is predictive of eventual wound closure [26,27].

### Statistical analysis

The wound assessment data collected from 59 HHAs were analyzed using the Statistical Package for Social Sciences [SPSS; IBM Corp, Armonk, New York. Version 29; 2024]. The analysis included conducting descriptive statistics for numeric variables such as age and categorical variables such as sex and various types of wounds. Summary statistics were illustrated as frequencies expressed in percentages (%) or as mean values and standard deviation (SD).

Bivariate analyses were performed to explore relationships between different variables. A sample *t-test* was employed to investigate whether there was a statistically significant mean difference in the average number of days required to heal wounds across different types of wounds when comparing the assessment years of 2023 and 2022. Additionally, this analysis aimed to compare the average reduction in wound area size, the average time taken to record improvement, and the age of patients for those diabetic ulcers (DUs) and pressure injuries (PIs) that were classified as non-healed but showed improvement in both 2022 and 2023. To ensure the statistical tests’ validity, the data’s normality was assessed using the Shapiro-Wilk test. The analyses showed that normal distribution of data as assessed by Shapiro-Wilk’s normality test (*P* > 0.05). Additionally the Levene test was utilized to evaluate the homogeneity of variances and there was homogeneity of variances, as assessed by Levene’s test for equality of variances for the assessed groups (*P* = 0.136, *P* = 0.221, respectively). A 95% confidence interval (CI) was reported, and the significance level was set at a *P* value of less than.05.

Additionally, subgroups were created to categorize healed wounds into two distinct groups based on the time elapsed between the first and last evaluations for wounds that were marked as healed. These groups were defined as wounds that healed in less than three months and those that healed in more than three months for the assessment years of 2022 and 2023. Furthermore, non-healed but improved PI and DU wounds were subcategorized based on their initial size, specifically those with an area of less than or equal to 4 cm² and those greater than 4 cm². For diabetic ulcers, the subcategories were based on initial sizes of less than or equal to 2 cm² and greater than 2 cm². A Chi-square test was conducted to compare the proportions of different types of healed wounds across the subgroups of healed wounds, as well as to analyze the percentage of improvement, sex, and acquisition of wounds for the improved non-healed wounds across the assessment years. The data were presented as frequencies expressed in percentages (%).

Two-way ANOVA tests were conducted to compare the average number of days required to heal different types of healed wounds across the subgroups of healed wounds (those healing in less than three months versus those healing in more than three months) during the assessment years.

Additionally, the average area size reduction, the average time taken to record improvement, and the age of patients were compared across the non-healed but improved PI and DU wounds. The results of these analyses were presented as mean values accompanied by their standard deviation.

Post hoc analyses involved conducting pairwise comparisons using multiple z-tests of two proportions, which were adjusted with a Bonferroni correction to account for multiple comparisons. This was done to test for significant changes between the different groups of input parameters. The significance level for the comparisons among these groups was set at a *P value* of <.0125.

## Results

### Overall characteristics

The data was collected from 195,915 wounds assessed at the participating HHAs using the technology in 2022 and 2023. Overall, the mean age of patients with wounds was 74.9 **± **15.9 years, with approximately half of the patients (47.9%) being female. In 2022, 66,878 wounds were assessed, and 129,040 wounds were assessed in 2023. The included wounds comprised various types, with surgical wounds accounting for 30.4% and pressure injuries for 18.1%, making them the most common.

In 2022, 20,128 (30%) of assessed wounds were healed, and 33,662 (26.1%) of assessed wounds were healed in 2023 (Table 1). The included wounds comprised various types, with surgical wounds accounting for 21.0% in 2022 and 35.9% in 2023, and pressure injuries accounting for 19.8% in 2022 and 17.0% in 2023, making them the most common wounds.

**Table 1:** Overall characteristics of wound records at adopted branches in 2022 and 2023.

	Adopted Branches 2022 Healed Episodes	Adopted Branches 2023 Healed Episodes
**Number of episodes managed at participating branches**	20,128 (30.0%)	33,662 (26.1%)
**Wound types**		
Arterial Ulcer	243 (1.2%)	307 (0.9%)
Burn	172 (0.9%)	268 (0.8%)
Diabetic Ulcer	1,019 (5.1%)	1,210 (3.6%)
Pressure Injury	3,994 (19.8%)	5,709 (17.0%)
Skin Tear	2,061 (10.2%)	3,080 (9.1%)
Surgical Wound	4,235 (21.0%)	12,090 (35.9%)
Venous	2,185 (10.9%)	2,904 (8.6%)
Other*	6,219 (30.9%)	8094 (24.0%)
**Sex**		
Male	10,488 (52.1%)	16,531 (49.1%)
Female	9,640 (47.9%)	17,000 (50.9%)
**Age (**Mean±SD)	74.9 ± 15.9	73.3 ± 14.7
**Sex (Females%)**	9,640 (47.9%)	17,000 (50.9%)
Arterial Ulcer	92 (37.9%)	136 (44.3%)
Burn	83 (48.3%)	145 (54.7%)
Diabetic Ulcer	358 (35.1%)	392 (32.7%)
Pressure Injury	2064(51.7%)	3050(54.4%)
Skin Tear	1,011(49.1%)	1,503(48.9%)
Surgical Wound	2116(50.0%)	6432(53.3%)
Venous	930(42.0%)	1342(46.4%)
**Age (**Mean±SD)	74.9 ± 15.9	73.3(±14.9)
Arterial Ulcer	75.3 ± 14.8	75.2(±13.2)
Burn	71.9 ± 15.1	68.7 ± 15.5)
Diabetic Ulcer	68.8 ± 13.8	68.1(±13.2)
Pressure Injury	76.8 ± 15.1	78.8(±14.3)
Skin Tear	83.6 ± 12.7	82.8 ± 11.7)
Surgical Wound	68.1 ± 14.9	67.1(±13.4)
Venous	77.0 ± 13.4	76.5(±13.4)

*Other Types of wounds: Abrasion, laceration, blisters, seroma, carcinoma, hematoma

Overall, there were no statistically significant differences (P > 0.05) between the distribution of wounds assessed or healed in 2022 and 2023, except for the surgical wounds, indicating a fairly comparable distribution of wound types across both years.

### Reduction in the average time to heal a wound

**Fig 2:**
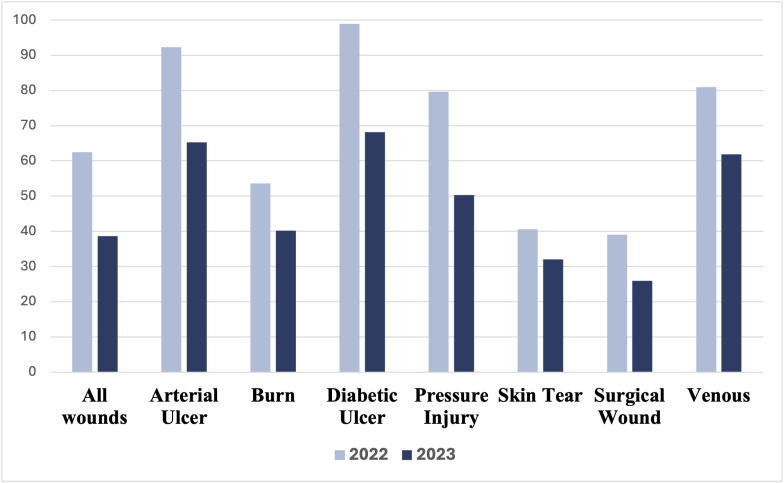
Average days (mean) to heal a wound in 2022 and 2023.

In 2023, the adoption branches saw a significant decrease in the average days required to heal a wound compared to 2022 (P < 0.001). On average, the adoption group experienced a reduction of 23.9 days per wound (equivalent to 3.4 weeks), with healing time decreasing from 62.5 days (8.9 weeks) in 2022 to 38.6 days (5.5 weeks) in 2023. This represents a 38.2% improvement in average healing time for the adoption branches (Table 2) (Fig 2).

**Table 2:** Average days to heal a wound in 2022 and 2023.

	Adopted Branches 2022 Average Days to Heal (N = 20,128) Mean ±SD	Adopted Branches 2023 Average Days to Heal (N = 33,662) Mean ±SD	P value	T value	95% CI of mean difference
**All wounds**	**62.5 ± 90.8**	**38.6 ± 47.6**	**<0.001**	34.63	22.7-25.1
Arterial Ulcer	92.3 ± 100.9	65.2 ± 65.8	**<0.001**	4.22	16.9-46.4
Burn	53.6 ± 80.8	40.2 ± 46.8	**0.044**	2.02	1.7-25.7
Diabetic Ulcer	98.9 ± 119.7	68.1 ± 65.5	**<0.001**	7.37	22.7-39.2
Pressure Injury	79.6 ± 114.2	50.3 ± 58.3	**<0.001**	17.85	31.2-39.8
Skin Tear	40.6 ± 58.8	32.0 ± 36.6	**0.046**	5.85	5.7-11.5
Surgical Wound	39.0 ± 58.1	25.9 ± 32.1	**<0.001**	13.86	11.2-14.9
Venous	80.9 ± 103.7	61.8 ± 65.5	**<0.001**	7.57	14.2-24.1

Additionally, significant differences were observed in the average days saved between 2022 and 2023 for various wound types. The highest reduction was observed in diabetic ulcers with 30.8 days (equivalent to 4.4 weeks), followed by pressure injuries with an average decrease of 29.3 days (4.2 weeks), and venous ulcers with 19.1 days (2.7 weeks) (P < 0.001) (Table 2).

### Comparison of proportions and time to heal for wounds healed in less than vs. more than three months

In 2023, the adoption branches saw an overall 8.9% increase in the proportion of wounds healed in less than three months compared to 2022. The most significant increases were observed in arterial ulcers (16.7%) and pressure injuries (11.2%), while burn wounds showed a slight non-significant decrease of 0.4% in the proportion of wounds healed in less than three months (Table 3).

**Table 3:** Proportion of healed wounds by healing duration in 2022 and 2023.

	Adopted Branches 2022 Healed wounds in <3 months (N = 16,284) (N (%)	Adopted Branches 2022 Healed wounds in >3 months (N = 3,844) (N (%)	Adopted Branches 2023 Healed wounds in <3 months (N = 20,128) (N (%)	Adopted Branches 2023 Healed wounds in >3 months (N = 33,662) (N (%)	P value (95% CI) Across groups
**All wounds**	16284(81%)	3844(19%)	30237(89.9%)	3425(10.2%)	**0.045 (0.032-0.058)**
Arterial Ulcer	151(62.1%)	92(37.9%)	242 (78.8%)	64 (21.2%)	**<0.001 (0.000-0.003)**
Burn	156(90.7%)	16(9.3%)	242 (90.3%)	26 (9.7%)	0.457 (0.426-0.488)
Diabetic Ulcer	687(67.4%)	332(32.6%)	881(72.8%)	329(27.2%)	0.053(0.039-0.067)
Pressure Injury	2,912(72.9%)	1082(27.1%)	4901 (84.1%)	908(15.9%)	**<0.001 (0.000-0.004)**
Skin Tear	1,860(90.2%)	201(9.8%)	2891(93.9%)	189(6.1%)	0.677 (0.0648-0.706)
Surgical Wound	3,843(90.7**%)**	392(9.3%)	11578 (95.8%)	512 (4.2%)	0.782 (0.756-0.808)
Venous	1,586(72.6%)	599(27.4%)	2276(78.4%)	628(21.6%)	0.651(0.621-0.681)

**Fig 3:**
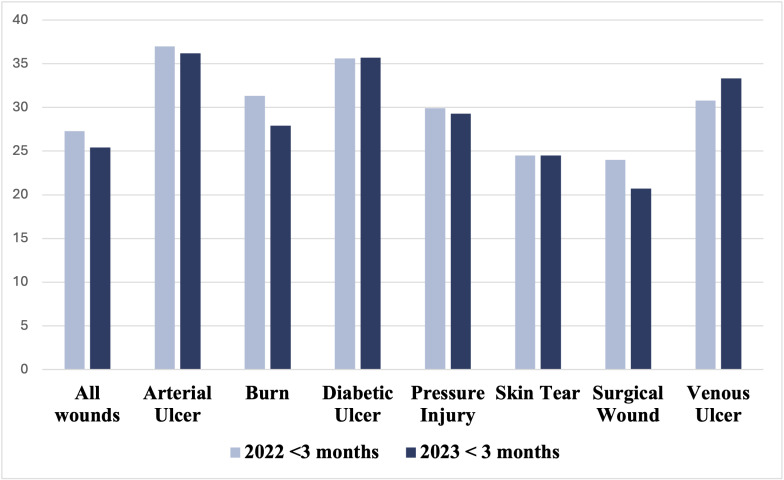
Comparison of mean days of wound healing across different wound types for wounds healed in less than 3 months durations (2022 and 2023).

Additionally, within the same timeframe, the average time taken for wounds to heal within these three months decreased, especially for burn and surgical wounds, which saw a reduction of 3.5 days (0.5 weeks) (Table 4). Fig 3

**Table 4:** Comparison of mean days of wound healing across different wound types by healing durations in 2022 and 2023.

	Adopted Branches 2022 Healed wounds in <3 months (N = 16,284) (Mean days±SD)	Adopted Branches 2022 Healed wounds in >3 months (N = 3,844) (Mean days±SD)	Adopted Branches 2023 Healed wounds in <3 months (N = 20,128) (Mean days±SD)	Adopted Branches 2023 Healed wounds in >3 months (N = 33,662) (Mean days±SD)	P value (95% CI) Across groups
**All wounds**	27.3 ± 21.0a	212.9 ± 117.0 b	25.4 ± 19.9a	155.3 ± 60.5 c	**0.014 (0.007-0.021)**
Arterial Ulcer	37.0 ± 23.5 a	195.3 ± 102.0 b	36.2 ± 23.9 a	173.1 ± 60.1 c	0.067 (0.052-0.082)
Burn	31.3 ± 23.0 a	274.5 ± 108.8 b	27.9 ± 19.2 a	154.7 ± 68.0 c	**<0.001 (0.000-0.005)**
Diabetic Ulcer	35.6 ± 23.8 a	230.1 ± 131.6 b	35.7 ± 23.7a	154.8 ± 61.2 c	**<0.001 (0.000-0.003)**
Pressure Injury	29.9 ± 22.8 a	234.5 ± 127.5 b	29.3 ± 21.6 a	161.3 ± 65.1 c	**<0.001 (0.000-0.007)**
Skin Tear	24.5 ± 17.7 a	189.8 ± 96.2 b	24.5 ± 17.8 a	146.4 ± 55.5 c	0.045 (0.0322-0.058)
Surgical Wound	24.0 ± 19.2 a	185.4 ± 96.4 b	20.7 ± 17.0 a	154.8 ± 61.2 b	0.054 (0.040-0.068)
Venous	30.8 ± 22.4a	213.6 ± 117.0 b	33.3 ± 22.9 a	161.3 ± 65.1 c	0.024(0.015-0.033)

*Significant difference using Post hoc analysis with a Bonferroni correction was accepted at *p* < .0125. Different letters between groups = significant difference(P < 0.0125). Same letters between groups = non-significant difference (P ≥ 0.0125).

**Fig 4:**
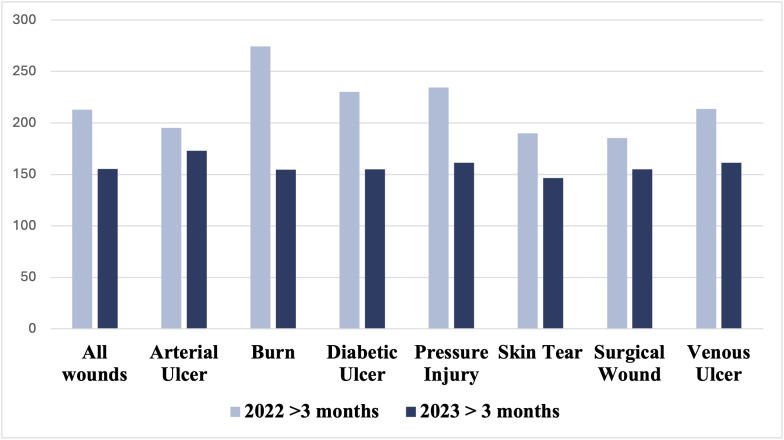
Comparison of mean days of wound healing across different wound types for wounds healed in more than 3 months durations (2022 and 2023).

Furthermore, for wounds that took more than three months to heal, there was a more significant decrease in the average time to heal, with an overall average of 57.6 days (8.2 weeks) saved (P = 0.014), resulting in 27% improvement. This was particularly notable for burn wounds (17.1 weeks), diabetic ulcers (10.8 weeks), pressure injuries (10.5 weeks), and venous ulcers (7.5 weeks) (Table 4) Fig 4.

### Key characteristics of non-healed but improved diabetic ulcers

In 2022, 45.3% of non-healed diabetic ulcer (DU) wounds showed an improvement in area size, compared to 29.5% in 2023. However, the average area size reduced in 2023 was greater (7.9 cm2) than in 2022 (4.4 cm^2^). The moderate Cohen’s effect size shows that 2023 had larger wound size reduction. There was a significant decrease in the average time to show improvement (P < 0.001), with 84.8 days (12.1 weeks) in 2022 compared to 62.8 days (9 weeks) in 2023, representing a 25.9% improvement. Additionally, there was a slight 1.2% increase in the proportion of acquired DUs that was improved from 2022 to 2023, although this was not statistically significant (Table 5).

**Table 5:** Key characteristics of non-healed DU wound records at adopted branches in 2022 and 2023.

	Adopted Branches 2022 Non-healed but Improved DU Episodes N = 1,356 (45.3%)	Adopted Branches 2023 Non-healed but Improved DU Episodes N = 1,520 (29.5%)*	P Value	Effect Size (Cohen’s d) [95% CI]
**Average area size reduction (cm**^**2**^)	4.4 ± 9.10	7.9 ± 7.3	**<0.001**	**-0.424 (-0.498,-0.350)**
**Average days to last improvement**	84.8 ± 112.5	62.8 ± 63.2	**<0.001**	**0.241 (0.168,0.315)**
**% of improvement**	55% ± 30.0%	54% ± 28.0%	0.150	0.034 (-0.039,0.108)
**Sex**				
Male	835 (61.6%)	1003 (66.0%)	0.234	
Female	521 (31.7%)	517 (34.0%)		
**Age (**Mean±SD)	67.2 ± 14.0	66.4 ± 14.8	0.354	
**Wound acquisition** Acquired (after admission)	226 (16.6%)	270 (17.8%)		
External (on admission)	1,130 (83.4%)	1,250 (82.2%)	0.067	

*Out of non-healed DU wounds (2,988 in 2022 and 5141 in 2023).

Overall, there were no statistically significant differences (P > 0.05) in the distribution of age, sex, percentage of improvement, and wound acquisition of the non-healed but improved DUs between 2022 and 2023 (Table 5).

### A comparison of non-healed but improved diabetic ulcers based on initial wound surface area size

In comparing 2022 and 2023, a significant difference was found in initial area sizes larger than 2 cm². The mean area size in 2023 was 10.2 cm², bigger than the 6.56 cm² average area recorded in 2022. No significant differences were observed for areas smaller than 2 cm². Additionally, the average time required for DU improvement decreased significantly for areas larger than 2 cm^2^ (P < 0.001), from 98.7 days (14 weeks) to 63.8 days (9.1 weeks), representing a 35.6% improvement in wound management efficiency. Overall, when examining the demographic factors, there were no statistically significant differences (P > 0.05) in the distribution of age or sex between the years 2022 and 2023, as illustrated in Table 6.

**Table 6:** Comparison of the Non-healed but Improved DUs Based on Initial Wound Area Size in 2022 vs. 2023.

	Adopted Branches 2022 Initial Area ≤ 2 cm^2^ N = 570 (42%)	Adopted Branches 2022 Initial Area >2 cm^2^ N = 786 (58%)	P value 2022 (<vs > 2 cm^2^)	Adopted Branches 2023 Initial Area ≤ 2 cm^2^ (N = 20,128)	Adopted Branches 2023 Initial Area >2 cm^2^ (N = 33,662)	P value 2023 (<vs > 2 cm^2^)	Overall P value between 2022 and 2023*
**Average area size reduction** (cm^2^)	0.51 ± 0.44 a	6.56 ± 11.3 c	**<0.001**	1.9 ± 1.5 a	10.2 ± 7.30 b	**<0.001**	**<0.001**
**Average days to last improvement**	65.1 ± 86.2 a	98.7 ± 126.4 b	**<0.001**	60.4 ± 66.9 a	63.8 ± 61.8 a	0.683	**<0.001**
**Improvement** %	50% ± 32.0% a	59% ± 30.0% b	**0.034**	45% ± 27.0% a	57% ± 28.0% b	**<0.001**	**<0.001**
**Sex** %							
Male	348 (61.0%)	387 (62.0%)	0.265	370 (62.5%)	627 (67.5%)	0. 241	0.162
Female	222 (39.0%)	399 (38.0%)		222 (37.5%)	301 (32.4%)		
**Age (**Mean±SD)	68.7 ± 12.7	65.4 ± 14.0	0.374	69.7 ± 13.1	67.8 ± 14.1	0.515	0.510

*Significant difference using Post hoc analysis with a Bonferroni correction was accepted at *p* < .0125. Different letters between groups = significant difference(P < 0.0125). Same letters between groups = non-significant difference (P ≥ 0.0125).

### Key characteristics of non-healed but improved pressure injuries

In 2022, the average area size of non-healed pressure injuries (PIs) decreased by approximately 5.2 cm². The positive trend continued in 2023, with the average reduction in area size increasing to 17.7 cm² for non-healed PIs, compared to 5.7 cm² in 2022. The overall improvement in PI area size also increased from 56% in 2022 to 61% in 2023, marking a 5% rise. Additionally, there was a significant decrease in the average duration required to observe improvement in these wounds. In 2022, the average time taken to show improvement was 86.5 days (equivalent to 12.3 weeks), which reduced to 64.5 days (or 9.2 weeks) in 2023, representing a 25.4% improvement. (Table 7). Effect size analysis revealed a moderate effect for area size reduction (Cohen’s d = -0.501, 95% CI [-0.549, -0.453]), indicating that wound reduction was significantly greater in 2023 compared to 2022. A small effect was observed for days to last improvement (Cohen’s d = 0.222, 95% CI [0.174, 0.269]) and % improvement (Cohen’s d = -0.175, 95% CI [-0.223, -0.128]), suggesting slight but meaningful differences between the two years.

**Table 7:** Key characteristics of non-healed PI at adopted branches in 2022 and 2023.

	Adopted Branches 2022 Non-healed but Improved DU Episodes N = 2576 (30.0%)	Adopted Branches 2023 Non-healed but Improved DU Episodes N = 5194 (30.4%) *	P Value	Effect Size (Cohen’s d) [95% CI]
**Average area size reduction** (cm^2^)	5.2 ± 10.4	17.7 ± 33.7	**<0.001**	**-0.501 (-0.549,-0.453)**
**Average days to last improvement**	86.5 ± 122.1	64.5 ± 69.3	**<0.001**	**0.222 (0.174, 0.269)**
**% of improvement**	56.0% ± 29.0%	61.0% ± 28.0%	**0.042**	**-0.175 (-0.223,-0.128)**
**Sex**				
Male	1,186 (46.1%)	2338 (45.1%)	0.211	
Female	1,388 (53.9%)	2,856 (54.9%)		
**Age (**Mean±SD)	74.5 ± 17.7	75.4 ± 15.8	0.362	
**Wound** acquisition				
**Acquired (after admission)**	578 (22.4%)	1,379 (26.5%)	0.178	
**External (on admission)**	1,998 (77.6%)	3,815 (73.5%)		

*Out of non-healed PI wounds (8576 in 2022 and 17084 in 2023).

### A comparison of non-healed but improved pressure injuries based on initial wound surface area size

When comparing the years 2022 and 2023, there was a significant difference in the initial area sizes exceeding 4 cm². The mean reduction in area size exceeding 4 cm² in 2023 was 16.2 cm², which was significantly larger than the average area size of 10.4 cm² in 2022. However, the changes in area size were mainly seen in the larger categories, with no significant differences noted for areas smaller than 4 cm². Additionally, the percentage of improvement for areas larger than 4 cm² increased by 3% from 2022 to 2023, although this change did not reach statistical significance.

In addition to the changes in area size, the average time required to observe improvement in PI reduced significantly for areas larger than 4 cm² (p-value < 0.001) from 101.8 days (14.5 weeks) in 2022 to 66.5 days (9.5 weeks) in 2023, marking a 34.7% improvement in the efficiency of wound management practices. Overall, when examining demographic factors such as age and sex, there were no statistically significant differences (P > 0.05) in their distribution between the years 2022 and 2023, as illustrated in Table 8.

**Table 8:** Comparison of the Non-healed but Improved PIs Based on Initial Wound Area Size in 2022 vs. 2023.

	Adopted Branches 2022 Initial Area ≤4 cm^2^ N = 1422 (55.2%)	Adopted Branches 2022 Initial Area >4 cm^2^ N = 1152 (44.8%)	P value 2022 (<vs > 4 cm^2^)	Adopted Branches 2023 Initial Area ≤4 cm^2^ N = 2,867(55.2%)	Adopted Branches 2023 Initial Area >4 cm^2^ N = 2,327(44.8%)	P value 2023 (<vs > 4 cm^2^)	Overall P value between 2022 and 2023*
**Average area size reduction** (cm^2^)	0.86 ± 0.82 a	10.4 ± 13.7 c	**<0.001**	1.57 ± 1.09 a	16.2 ± 32.8.2 b	**<0.001**	**<0.001**
**Average days to last improvement**	74.3 ± 109.6 a	101.8 ± 134.4 b	**<0.001**	48.2 ± 70.6 c	66.5 ± 70.6 d	**<0.001**	**<0.001**
**Improvement** %	54% ± 27.0% a	59% ± 30.0% b	**0.046**	49% ± 27.0% a	62% ± 28.0% b	**<0.001**	0.052
**Sex** %							
Male	616 (43.3%)	570 (49.4%)	0.265	1252 (43.6%)	1086 (46.6%)	0. 061	0.162
Female	806 (56.7%)	582 (50.5%)		1,615 (56.3%)	1,241 (53.3%)		
**Age (**Mean±SD)	76.8 ± 14.8	71.6 ± 20.3	0.374	78.9 ± 13.1	74.9 ± 15.9	0.425	0.510

* *Significant difference using Post hoc analysis with a Bonferroni correction was accepted at *p* < .0125. Different letters between groups = significant difference(P < 0.0125). Same letters between groups = non-significant difference (P ≥ 0.0125).

## Discussion

The findings of this quality improvement study illustrate significant insights into the potential benefits of incorporating DWCS into the standard wound care practices of HHA on wound healing outcomes. By analyzing a robust dataset comprising 195,915 wound assessments over a two-year period of the cross-sectional sample of 59 HHAs that adopted the digital wound care solution at the same year, this study demonstrated a significant improvement in the efficiency of wound healing processes. Notably, the data not only highlighted an overall enhancement in healing times but also offered a detailed analysis of DWCS impacted different types and sizes of wounds.

### Improvement in healing times

The study showed an overall reduction in the average healing times of 23.9 days (equivalent to 3.4 weeks) across various wound types from 2022 to 2023, representing a 38.2% improvement.

The results support previous research that emphasizes the benefits of digital health technologies in improving healing times [20,28]. This finding underscores the effectiveness of the DWCS in improving the wound healing process. Digital solutions often offer real-time access to secure, comprehensive, detailed wound assessment data. Furthermore, digital tools accurately assess wounds compared to other methods [25]. Their precise measurement of wounds’ area sizes and active tracking of size changes and surrounding wound bed areas [29] enhance wound monitoring and facilitate more consistent and evidence-based wound care practices, potentially leading to positive clinical outcomes [30].

Additionally, our study demonstrated a decrease in the average healing times across different wound types. For example, the study recorded a significant decrease of 30.8 days (equivalent to 4.4 weeks) for diabetic ulcers, 29.3 days (4.2 weeks) for PIs, and 19.1 days (2.7 weeks) for VUs.

These findings suggest that the DWCS could significantly benefit these complex wound types, often requiring more comprehensive management approaches. This coincides with previous research highlighting the benefits of digital health technologies in enhancing healing times for complex wounds, such as pressure injuries at their different stages in Skilled Nursing Facilities (SNFs) [20].

As many wounds are diverse and complex, so with active monitoring and proper assessment, clinicians can deliver personalized care-based strategies to wounds with unique characteristics [31], enhance treatment strategies to achieve faster healing [32], and control wound complications such as infection, ultimately leading to shorter healing duration and enhanced overall quality of care for patients.

This finding has an important financial implication for the healthcare system. Previous studies have indicated that shorter healing times of wounds could potentially reduce healthcare costs [32,33,34]. Studies have shown that nurses spend roughly 60% of their time changing dressings [35,36]. By utilizing advanced wound care technology to reduce healing time, the cost of wound management could be reduced by one-third compared to the expenses associated with additional clinic visits, wound supplies, and interventions [37]. Additionally, Lutz and colleagues, 2020) found that saving an average of 5 days in wound healing could save up to $5,000 per patient [34].

### Impact of DWCS on the proportion and healing duration of wounds healed in less than three months and more than three months

The higher proportion of wounds that healed within three months compared to those that healed in more than three months, along with the significant increase in this proportion from 2022 to 2023 (P = 0.045) and the 8.9% decrease in the number of wounds healed beyond three months, all underscore the potential impact of DWCS on enhancing both short and long-term wound management and efficiency. The analysis of these time groupings supports that DWCS benefit both acute and chronic wound healing, meaning technology enabled wound care should not be limited after delayed wound healing occurs.

The decrease in healing duration for wounds that took longer than three months to heal was particularly notable for chronic burn wounds (17.1 weeks), diabetic ulcers (10.8 weeks), PIs (10.5 weeks) and venous ulcers (7.5 weeks). This further underlines the potential for the DWCS to address specific challenges associated with chronic wound management effectively.

This observation aligns with the findings of Wang and colleagues in 2022, who demonstrated that the use of AI in wound care significantly reduced healing times for chronic diabetic ulcers and pressure injuries. This improvement was attributed to AI tools’ ability to provide precise wound assessments and optimized treatment recommendations [31].

Managing chronic wounds presents significant challenges for healthcare professionals and systems. Treating persistent wounds over a prolonged period often leads to clinician fatigue and attrition, contributing to stress and dissatisfaction [38].

Resource limitations, such as insufficient staffing and equipment, especially in a system that struggles with growing demand and high patient volumes, further complicate this issue, intensifying the strain on medical practitioners and patient care [39]. Moreover, the economic impact on healthcare systems is substantial; long-term wound treatment leads to increased costs for direct interventions and associated complications [40]. Research indicates that these expenses significantly affect healthcare budgets due to the extensive use of medical supplies, recurrent patient consultations, and supplementary procedures [41].

Therefore, by reducing the average healing period, clinicians can significantly reduce the time spent managing chronic wounds and applying dressings. A study by Mohammed et al. in 2024 revealed that implementing new wound assessment technology could save up to 44,808 hours of clinician time on PI dressing changes in a year [20]. This saved time could then be allocated to other patient care activities and enhance treatment plans, potentially improving overall healthcare delivery and patient outcomes [20].

Thus, healing patients faster, especially for chronic wounds, aligns with DWCS’s goal of optimizing patient recovery and potentially ensuring efficient resource utilization and reduced cost. These combined benefits can reduce the burden on the healthcare system.

### Non-healed but improved wounds

The study’s findings on non-healed but improved diabetic ulcers and pressure injuries provide an additional layer to the effectiveness of the DWCS. Although the proportion of non-healed diabetic ulcers showing improvement decreased from 45.3% in 2022 to 29.5% in 2023, the larger improvement of 44% for DU in average wound area size and the decreased time to heal by 25% in 2023 indicate that the DWCS enhances the management of these challenging cases. Additionally, the improvement in pressure injuries, with a notable increase in the average area size reduction of 70% and a significant decrease in the time to improvement of 25%, further reflects the positive impact of the DWCS on wound care outcomes.

These results suggest that even in cases where complete healing is not achieved, the DWCS can still facilitate meaningful progress in wound management. This is particularly important for patients with chronic wounds, as any reduced wound size and improved condition can lead to a better quality of life and reduced risk of complications. These findings are likely attributable to the DWCS’s monitoring of wound healing progress over time and its comprehensive documentation features. This enables clinicians to identify trends and patterns, guiding subsequent treatment strategies. The data-centric solution enhances the understanding of wound healing dynamics and supports the development of targeted interventions to address specific challenges encountered by patients with non-healed wounds.

The findings are in line with the studies conducted by [42,43,44]. Toh and colleagues observed significant improvements in wound area measurements and patient comfort using AI-powered digital wound assessment systems despite incomplete closure and healing of the wounds [42]. Similarly, Patel and colleagues highlighted that even for PI patients who did not achieve complete healing, the AI tools lead to meaningful advancements in PI patients’ wound state and overall quality of life [44]. Furthermore, Davis and coauthors demonstrated that AI tools enhanced diabetic ulcer management by providing accurate measurements and illustrating healing patterns, allowing clinicians to offer tailored treatment strategies and achieve considerable reductions in wound size and associated symptoms, even without achieving full closure [43].

### Non-healed but improved wounds based on initial wound size

The findings of wound improvement in non-healed DU and PI stratified by their initial size suggest that the DWCS has been effective for larger wound management, as significant reductions in area size and time to improve a wound were observed for DU and PI with initial sizes greater than 2 cm² and 4 cm², respectively. This finding is important as larger wounds often present more significant challenges in wound management and require prolonged healing periods [45]. Studies indicated that large-sized wounds often require more intensive and prolonged treatment protocols since they correlate with higher infection and complication rates [45].

Additionally, Sullivan et al’s study emphasized that larger chronic wounds, including diabetic foot ulcers, often encounter additional challenges to healing, such as compromised blood flow and an increased risk of comorbidities [46]. Similarly, Lantier and coauthors demonstrated that wounds exceeding 10 cm² often necessitate advanced, multifaceted treatment approaches, including specialized dressings and more frequent clinical interventions [47].

The DWCS’s capacity to support adequate progress in larger wounds underscores its potential to enhance the treatment of patients with complex, extensive wounds. Its effectiveness in managing larger chronic wounds can lead to a more comprehensive approach to wound care within healthcare systems. By incorporating the DWCS into standard protocols, healthcare providers can guarantee that patients with more extensive and complex wounds receive the essential attention and resources, thereby improving patient comfort. This integration can potentially sustain ongoing improvement in wound care practices, ultimately benefiting a broader wound patient population.

### Ethical consideration of AI in wound care

With the broad-scale incorporation of AI wound care technology, ethical concerns must correspondingly be addressed in order to ensure fidelity to its implementation. Data privacy and security being a major player since AI rationalization hugely relies on extensive data collectivity and digital monitoring, patients’ sensitive clinical information should be particularly handled with extreme diligence [48]. Maintaining transparency in AI decisions, clinician surveillance, and aligning with recommendations supported by evidence-based guidelines are other notable factors necessary for the development of faith and stimulation of equitable health delivery [49]. Future research should focus on strategies for mitigating bias, explainability of AI recommendations, and common ethics frameworks that provide guidance on the appropriate use of AI in wound care.

### Limitations

The study employed a descriptive design, making it difficult to establish a causal relationship between integrating DWSC in practice and the observed improvement in wound healing. While the study showed a significant reduction in healing times and the higher percentage of wounds healed in under three months, attributing these outcomes solely to the DWCS necessitates consideration of other potential contributing factors, like individual patient characteristics and wound complexity and severity. Future studies should incorporate randomized controlled trail or quasi-experimental design with a comparative group to assess causality.

A major strength of this study is its assessment of sustained impact over two years, providing a more complete overview of long-term effects following DWCS adoption. Yet, selection bias may present a limitation, as HHAs involved in the study adopted DWCS voluntarily. This could imply that these agencies were more likely to adopt advanced clinical workflow and innovative ideas. Staff in these agencies may have had greater engagement with training programs and wound care protocols compared to non-participating HHAs, thereby limiting the generalizability of findings. Future studies should include a comparative assessment alongside HHAs not participating in DWCS adoption for better isolation of their effects.

Another likely source of bias is the so-called Hawthorne effect; healthcare practitioners may have enhanced their documentation and adherence to clinical instructions just because they were aware that they were being evaluated on their practices relating to wound care. While the sustained improvement over two years was captured by this study, future research should focus on whether these improvements remain stable with the sustained use over the years.

Although the study controlled for certain demographic factors like age and sex and examined various wound types, other patient-specific variables such as comorbidities, overall health, and wounds’ complexity and severity might still contribute to the healing process. The study’s data did not include detailed patient health information, wound severity or adherence to treatment plans; therefore, for a more comprehensive understanding of the effectiveness of the DWCS, future investigations should take these patient-related factors and wound severity into account.

Inclusion of such factors in future analyses would further strengthen the findings and provide a better understanding of how certain patient characteristics affect wound assessment and treatment responses

Additionally, the study analyzed data from various HHAs that started using the solution around the same time, some of which may have employed different clinical practices and wound care protocols. Discrepancies in applying DWCS in practice, adherence to technology protocols, and variations in care practices could impact the consistency of the results and potentially affect the generalizability of the findings. Research that accounts for these differences and investigates the impact of standardized protocols and adherence to DWSC across different settings would provide more reliable insights.

Furthermore, the study assessed the effects of the DWCS on wound healing times and rates over a two-year period. However, it’s important to explore the long-term sustainability of these improvements over extended periods. Future research should also investigate each complex wound type individually and conduct long-term longitudinal studies to better understand how the DWCS impacts each type’s improvement over time.

## Conclusion

The study reported a significant improvement of 38% in time to heal a wound with the sustainable use of DWCS from 2022 to 2023 for different types of wounds across a cohort of 59 HHAs. This improvement highlights the potential impact of DWCS on accelerating the wound healing process. Furthermore, the observed significant decrease in healing duration for chronic wounds for diabetic ulcers, pressure injuries, and burn wounds underscores the association between the utilization of DWCS and efficiency in managing chronic wounds and addressing challenges associated with them. Furthermore, the DWCS has shown promise in managing larger wounds efficiently, as significant reductions in area size and time to improve a wound were observed for DU and PI with larger initial area sizes. addressing longstanding challenges in wound care. Overall, these findings support DWCS’s important role in improving wound care outcomes, which ultimately can optimize resource use, and enhance patient care delivery. As healthcare providers continue to integrate technological advancements into practice, it is essential to vigilantly monitor and evaluate the impact of these innovations on patient health and overall healthcare delivery. Future research should explore the impact of implementing wound care technology on complex, severe wounds and delve into the long-term impact in various healthcare settings. Further longitudinal studies and randomized controlled trials (RCTs) should be conducted to examine the sustained effectiveness of DWCS over extended periods across different settings. These studies should assess whether the initial benefits observed in wound healing rates remain consistent beyond two years, how clinician adherence to DWCS changes over time, and whether the system continues to optimize wound management across diverse clinical environments. Moreover, future studies should focus on conducting economic evaluations to comprehensively understand the financial impact of implementing DWCS on the healthcare system. These analyses should examine how reductions in wound healing time translate into cost savings after considering the initial investment and ongoing operational costs of DWCS.

## References

[1] Sen CK. Human wounds and its burden: an updated compendium of estimates. Adv Wound Care (New Rochelle). 2019;8(2):39–48. doi: 10.1089/wound.2019.0946 30809421 PMC6389759

[2] Chen L, Cheng L, Gao W, Chen D, Wang C, Ran X. Telemedicine in chronic wound management: systematic review and meta-analysis. JMIR Mhealth Uhealth. 2020;8(6):e15574. doi: 10.2196/15574 32584259 PMC7381084

[3] American Diabetes Association. Improving care and promoting health in populations: standards of medical care in diabetes-2021. Diabetes Care. 2021;44(Suppl 1):S7–14. doi: 10.2337/dc21-S001 33298412

[4] Armstrong DG, Tan T-W, Boulton AJM, Bus SA. Diabetic foot ulcers: a review. JAMA. 2023;330(1):62–75. doi: 10.1001/jama.2023.10578 37395769 PMC10723802

[5] Al-Mohaithef M, Abdelmohsen SA, Algameel M, Abdelwahed AY. Screening for identification of patients at high risk for diabetes-related foot ulcers: a cross-sectional study. J Int Med Res. 2022;50(3):3000605221087815. doi: 10.1177/03000605221087815 35343272 PMC8966102

[6] Sears P, Crook DW, Louie TJ, Miller MA, Weiss K. Fidaxomicin attains high fecal concentrations with minimal plasma concentrations following oral administration in patients with Clostridium difficile infection. Clin Infect Dis. 2012;55 Suppl 2(Suppl 2):S116-20. doi: 10.1093/cid/cis337 22752859 PMC3388019

[7] Clayton W Jr, Elasy TA. A review of the pathophysiology, classification, and treatment of foot ulcers in diabetic patients. Clinical Diabetes. 2009;27(2):52–8. doi: 10.2337/diaclin.27.2.52

[8] Akkus G, Sert M. Diabetic foot ulcers: a devastating complication of diabetes mellitus continues non-stop in spite of new medical treatment modalities. World J Diabetes. 2022;13(12):1106–21. doi: 10.4239/wjd.v13.i12.1106 36578865 PMC9791571

[9] Bonkemeyer Millan S, Gan R, Townsend PE. Venous ulcers: diagnosis and treatment. Am Fam Physician. 2019;100(5):298–305. 31478635

[10] Li Z, Lin F, Thalib L, Chaboyer W. Global prevalence and incidence of pressure injuries in hospitalised adult patients: a systematic review and meta-analysis. Int J Nurs Stud. 2020;105:103546. doi: 10.1016/j.ijnurstu.2020.103546 32113142

[11] Lindholm C, Searle R. Wound management for the 21st century: combining effectiveness and efficiency. Int Wound J. 2016;13 Suppl 2(Suppl 2):5–15. doi: 10.1111/iwj.12623 27460943 PMC7949725

[12] Ruedrich ED, Henzel MK, Hausman BS, Bogie KM. Reference gene identification for reverse transcription-quantitative polymerase chain reaction analysis in an ischemic wound-healing model. J Biomol Tech. 2013;24(4):181–6. doi: 10.7171/jbt.13-2404-003 24294111 PMC3792703

[13] Stephen-Haynes J, Bielby A, Searle R. Putting patients first: reducing the human and economic costs of wounds. Wounds UK. 2011;7:47–55.

[14] Carter MJ. Why is calculating the “True” cost-to-heal wounds so challenging? Adv Wound Care (New Rochelle). 2018;7(11):371–9. doi: 10.1089/wound.2018.0829 31768298 PMC6874809

[15] Monika P, Chandraprabha MN, Rangarajan A, Waiker PV, Chidambara Murthy KN. Challenges in healing wound: role of complementary and alternative medicine. Front Nutr. 2022;8:791899. doi: 10.3389/fnut.2021.791899 35127787 PMC8811258

[16] Nagle SM, Stevens KA, Wilbraham SC. Wound Assessment. In: StatPearls [Internet]. Treasure Island (FL): StatPearls Publishing; 2024 Jan-. Updated 2023 Jun 26. Available from: https://www.ncbi.nlm.nih.gov/books/NBK482198/. 29489199

[17] Ousey K, Gilchrist B, James H. Understanding clinical practice challenges: a survey performed with wound care clinicians to explore wound assessment frameworks. Wounds Int. 2018;9(4):58–62.

[18] Ding S, Lin F, Gillespie BM. Surgical wound assessment and documentation of nurses: an integrative review. J Wound Care. 2016;25(5):232–40. doi: 10.12968/jowc.2016.25.5.232 27169338

[19] Palese A, Luisa S, Ilenia P, Laquintana D, Stinco G, Di Giulio P, et al. What is the healing time of Stage II pressure ulcers? Findings from a secondary analysis. Adv Skin Wound Care. 2015;28(2):69–75. doi: 10.1097/01.ASW.0000459964.49436.ce 25608012

[20] Mohammed HT, Mannion D, Cassata A, Fraser RD. Trends in pressure injury prevalence rates and average days to healing associated with adoption of a comprehensive wound care program and technology in skilled nursing facilities in the United States. Wounds. 2024;36(1):23–33. doi: 10.25270/wnds/23089 38417821

[21] Kwame A, Petrucka PM. A literature-based study of patient-centered care and communication in nurse-patient interactions: barriers, facilitators, and the way forward. BMC Nurs. 2021;20(1):158. doi: 10.1186/s12912-021-00684-2 34479560 PMC8414690

[22] Barakat-Johnson M, Jones A, Burger M, Leong T, Frotjold A, Randall S, et al. Reshaping wound care: evaluation of an artificial intelligence app to improve wound assessment and management amid the COVID-19 pandemic. Int Wound J. 2022;19(6):1561–77. doi: 10.1111/iwj.13755 35212459 PMC9111327

[23] Mohammed HT, Bartlett RL, Babb D, Fraser RDJ, Mannion D. A time motion study of manual versus artificial intelligence methods for wound assessment. PLoS One. 2022;17(7):e0271742. doi: 10.1371/journal.pone.0271742 35901189 PMC9333325

[24] Wang SC, Anderson JAE, Evans R, Woo K, Beland B, Sasseville D, et al. Point-of-care wound visioning technology: Reproducibility and accuracy of a wound measurement app. PLoS One. 2017;12(8):e0183139. doi: 10.1371/journal.pone.0183139 28817649 PMC5560698

[25] Alonso MC, Mohammed HT, Fraser RD, Ramirez Garcia Luna JL, Mannion D. Comparison of wound surface area measurements obtained using clinically validated artificial intelligence-based technology versus manual methods and the effect of measurement method on debridement code reimbursement cost. Wounds. 2023;35(10):E330–8. doi: 10.25270/wnds/23031 37956346

[26] Sheehan P, Jones P, Caselli A, Giurini JM, Veves A. Percent change in wound area of diabetic foot ulcers over a 4-week period is a robust predictor of complete healing in a 12-week prospective trial. Diabetes Care. 2003;26(6):1879–82. doi: 10.2337/diacare.26.6.1879 12766127

[27] Hudacko RM, Manoukian AV, Schneider SH, Fyfe B. Clinical resolution of glycogenic hepatopathy following improved glycemic control. J Diabetes Complications. 2008;22(5):329–30. doi: 10.1016/j.jdiacomp.2007.11.004 18413180

[28] Zhang Z, Li S, Zhang S. AI-based wound assessment: improving the precision and efficiency of wound care. Int J Artif Intell Res. 2020;35(4):785–94. doi: 10.1016/j.ijair.2020.04.005

[29] Comino-Sanz IM, Cabello Jaime R, Arboledas Bellón J, Jiménez-García JF, Muñoz-Conde M, Díez Requena MJ, et al. A digital tool for measuring healing of chronic wounds treated with an antioxidant dressing: a case series. Int J Environ Res Public Health. 2023;20(5):4147. doi: 10.3390/ijerph20054147 36901155 PMC10002323

[30] Biala KY. Case conferencing for wound care patients. Home Healthc Nurse. 2002;20(2):120–5; quiz 126. doi: 10.1097/00004045-200202000-00010 11839978

[31] Wang H, Chen J, Liu Y. Enhancing chronic wound management with artificial intelligence: a review of recent advances. IEEE Rev Biomed Eng. 2022;15:216–26. doi: 10.1109/RBME.2021.307418

[32] Le DTP, Pham TD. Unveiling the role of artificial intelligence for wound assessment and wound healing prediction. Exploration of Medicine. 2023;:589–611. doi: 10.37349/emed.2023.00163

[33] Abatangelo G, Marcucci M, Ravagnan G. Cost-effectiveness of advanced wound care technologies in chronic wound management. J Wound Care. 2021;30:1–10.

[34] Karim AS, Liu A, Lin C, Uselmann AJ, Eliceiri KW, Brown ME, et al. Evolution of ischemia and neovascularization in a murine model of full thickness human wound healing. Wound Repair Regen. 2020;28(6):812–22. doi: 10.1111/wrr.12847 32686215 PMC8592059

[35] O’Keeffe M. Evaluation of a community-based wound care programme in an urban area. Poster Presented at: EWMA Conference. Prague, Czech Republic; 2006.

[36] Royal College of Nursing. Frontline First: Nursing on Red Alert. London: The Royal College of Nursing; 2013. Accessed on Sept 9, 2024 Available from: http://www.rcn.org.uk/__data/assets/pdf_file/0003/518376/004446.pdf

[37] Abatangelo G, Marcucci M, Ravagnan G. Cost-effectiveness of advanced wound care technologies in chronic wound management. J Wound Care. 2021;30(3):187–96. doi: 10.12968/jowc.2021.30.3.187

[38] Smith J, Brown L, Taylor P. Impact of chronic wound management on clinician well-being: addressing fatigue and attrition. J Wound Care. 2022;31(5):450–7. doi: 10.12968/jowc.2022.31.5.450

[39] Baird M, O’Donnell JM, Martsolf GR. Effects of opting-out from federal nurse anesthetists’ supervision requirements on anesthesiologist work patterns. Health Serv Res. 2020;55(1):54–62. doi: 10.1111/1475-6773.13245 31835283 PMC6981044

[40] Johnson M, Smith T, Williams R. Economic impact of chronic wound management on healthcare systems. J Health Econ. 2021;45(2):123–31. doi: 10.1016/j.jhealeco.2021.01.005

[41] Korsholm M, Sørensen J, Mogensen O, Wu C, Karlsen K, Jensen PT. A systematic review about costing methodology in robotic surgery: evidence for low quality in most of the studies. Health Econ Rev. 2018;8(1):21. doi: 10.1186/s13561-018-0207-5 30194567 PMC6128948

[42] Toh KY, Wong CH, Yap SH. The role of artificial intelligence in managing chronic wounds: a review. J Wound Care. 2021;30(4):234–45. doi: 10.12968/jowc.2021.30.4.23433729841

[43] Davis MR, Singh A, Patel V. Enhancing diabetic ulcer management with AI: insights and outcomes. Diabetes Technol Ther. 2022;24(2):150–9. doi: 10.1089/dia.2021.0206

[44] Patel N, Kumar A, Clarke H. AI in pressure injury management: improving wound care outcomes beyond full healing. Int J Wound Manag. 2023;30(1):45–54. doi: 10.1111/ijwm.12753

[45] Zimmet P, Mikhail M, Greenfield S. The challenges of managing large wounds: implications for clinical practice. J Wound Care. 2021;30(6):342–50. doi: 10.12968/jowc.2021.30.6.342

[46] Sullivan N, Reddy S, Carlson H. The impact of wound size on healing time and management strategies in chronic wounds. Int Wound J. 2023;20(1):65–74. doi: 10.1111/iwj.12890

[47] Tsunoi Y, Sato N, Nishidate I, Ichihashi F, Saitoh D, Sato S. Burn depth assessment by dual-wavelength light emitting diodes-excited photoacoustic imaging in rats. Wound Repair Regen. 2023;31(1):69–76. doi: 10.1111/wrr.13056 36177703

[48] Mittelstadt BD, Allo P, Taddeo M, Wachter S, Floridi L. The ethics of algorithms: mapping the debate. Big Data & Society. 2016;3(2). doi: 10.1177/2053951716679679

[49] Bouderhem R. Shaping the future of AI in healthcare through ethics and governance. Humanit Soc Sci Commun. 2024;11(1). doi: 10.1057/s41599-024-02894-w

