# Expression of Concern: Impact of digital wound care solution on healing time: A descriptive study in home health settings

**DOI:** 10.1371/journal.pdig.0001025

**Published:** 2025-09-30

**Authors:** The PLOS Digital Health Editors

The *PLOS Digital Health* Editors issue this Expression of Concern due to concerns identified after publication of this article [1] about compliance with data-related requirements specified in the terms of the study’s ethics review exemption.

Additionally, the article was republished on June 5, 2025, to address an error. Please download this article again to view the correct version.

The article’s Data Availability statement is updated to: All relevant data are within the paper and its Supporting Information files.

Supporting Information files S1 and S2 Data are provided with this notice.

## Supporting information

S1 Data Healing time. (ZIP)

S2 Data Improved wounds. (ZIP)
